# Habitual Physical Activity and Sleep Duration in Institutionalized Older Adults

**DOI:** 10.3389/fneur.2021.706340

**Published:** 2021-07-27

**Authors:** Oliver Vogel, Daniel Niederer, Jan Wilke, Inaam El-Rajab, Lutz Vogt

**Affiliations:** Department of Sports Medicine, Goethe-University Frankfurt, Frankfurt, Germany

**Keywords:** accelerometry, Pittsburgh Sleep Quality Index, nursing home, old age health, sleep quality

## Abstract

**Background:** Physical activity and sleep quality are both major factors for improving one's health. Knowledge on the interactions of sleep quality and the amount of physical activity may be helpful for implementing multimodal health interventions in older adults.

**Methods:** This preliminary cross-sectional study is based on 64 participants [82.1 ± 6.4 years (MD ± SD); 22 male: 42 female]. The amount of physical activity was assessed by means of an accelerometer (MyWellness Key). Self-reported sleep parameters were obtained using the Pittsburgh Sleep Quality Index. The Barthel Index was used for physical disability rating. Bivariate correlations (Spearman's Rho) were used to explore relationships between the amount of physical activity and sleep quality. To analyse differences between categorial subgroups univariate ANOVAs were applied; in cases of significance, these were followed by Tukey-HSD *post-hoc* analyses.

**Results:** No linear association between physical activity and sleep quality was found (*r* = 0.119; *p* > 0.05). In subgroup analyses (*n* = 41, Barthel Index ≥90 pts, free of pre-existing conditions), physical activity levels differed significantly between groups of different sleep duration (≥7 h; ≥6 to <7 h; ≥5 to <6 h; <5h; *p* = 0.037).

**Conclusion:** There is no general association between higher activity levels and better sleep quality in the investigated cohort. However, a sleep duration of ≥5 to <6 h, corresponding to 7.6 h bed rest time, was associated with a higher level of physical activity.

## Background

Regular physical activity of a sufficient extent provides a broad range of health benefits. Sufficient physical activity is defined as weekly 150 min of moderate activity, or 75 min of vigorous activity, or a combination of both (1–4). Besides the generally recommended strengthening exercises twice per week, older adults (≥65 years) are also advised to include balance exercises. Physical activity is able to reduce the risk of various non-communicable diseases such as diabetes, cancer, osteoporosis, depression, cardiovascular diseases and metabolic diseases (5, 6). Moreover, sufficient physical activity enhances mobility and the ability to perform activities of daily living (ADLs) in older adults whilst also reducing the risk of falls and delaying, or preventing, care dependency (7). As a measure of independency, the Barthel Index rates the ability to perform ADLs as well as physical activity (8, 9).

Another major contributing factor to health and well-being in older adults is sleep quality. Good sleep impacts on the regeneration of the immune system and the metabolic brain processes (10–13). As well as throughout life, sleep quality determines the quality of health at higher age to a certain extent (14). However, sleep duration as one component of sleep quality decreases with increasing age, while the sleep itself shows augmented interruptions (15). Periods of deep sleep and Rapid-Eye-Movement (REM) sleep decline proportionally to the overall sleep duration. Sleep efficiency diminishes due to rising sleep latency and more frequent interruptions of sleep at old age (15). In nursing homes more than half of all inhabitants suffer from sleep disturbances (16).

Even though physical activity and sleep quality both contribute to the status of health, the underlying physiological mechanisms differ greatly. Nevertheless, there appear to be clinically important mutual relationships between these two factors (17). Therefore, changes in one factor may affect the health outcomes of both. A deeper understanding of the association between sleep parameters and physical activity may lead to advanced health promotion. It has yet to be clarified whether healthy doses of sleep and activity, as well as their health benefits, are combinable or if they possibly interfere.

Previous studies have examined associations of regular and acute bouts of physical activity with sleep parameters (18–21). Although a great variety of investigated types of physical activities and sleep parameters have led to an equally numerous variety of outcomes, there exists a consensus among the literature that physical activity increases sleep quality (22). As a crosscheck for the above-mentioned outcomes, sleep disturbances were associated with a limitation in physical activity in a cohort of cancer patients (23). However, in the case for sleep duration, there appears to be disagreement among the literature on the arguable association with physical activity (24–26).

Therefore, investigating the relation between physical activity and sleep parameters such as sleep duration may add to the current state of knowledge. A deeper understanding of the relation between these two factors might contribute to future research on their mutual health effects. Older adults generally exhibit decreased sleep quality and physical activity. Furthermore, the urgency of geriatric health promotion increases along with demographic change. Hence, the present study investigates the association between habitual physical activity and sleep parameters in older adults.

## Methods

### Ethical Standards and Study Design

This preliminary cross-sectional study was approved by the local review board (Goethe University, Department of Psychology and Sports Sciences, Ethics Committee; Approval-Number 2019-22) and conducted in accordance to the Declaration of Helsinki. Start date was in 2019. After agreement from the participating institutions' managements, the study design was presented at residents' meetings prior to recruitment *via* personal contact. All participants signed informed consent before inclusion.

### Participants

Residents (males and females) of nursing homes, assisted living institutions and senior residences aged over 65 were included. Exclusion criteria comprised acute infections or injuries, being bedridden and dementia [Montreal Cognitive Assessment—MoCA <17 (27)]. Participants were recruited by personal contact within institutions interested in the study. Overall, 64 older adults participated in the study (Table 1).

**Table 1:** Participants' characteristics.

	***N***	**%**
Female	42	65.6
Male	22	34.4
Total	64	100
	***M***	**SD**
Age (years)	82.1	6.3
Height (cm)	167.6	8.9
Weight (kg)	68.4	13.6
BMI (kg/m^2^)	24.3	3.9
Barthel-Index^1^ (points)	89.7	16.8
PSQI Total^2^ (points)	7.3	4.6
PSQI Component 1: Sleep Quality^3^ (points)	1.1	0.8
PSQI Component 2: Sleep latency^3^ (points)	1.2	1.1
PSQI Component 3: sleep duration^3^ (points)	1.0	1.1
PSQI Component 4: sleep efficiency^3^ (points)	1.1	1.2
PSQI Component 5: Sleep disturbances^3^ (points)	1.3	0.6
PSQI Component 6: Sleeping pills^3^ (points)	0.6	1.1
PSQI Component 7: Daytime sleepiness^3^ (points)	0.7	0.7
habitual physical activity^4^ (meth/week)	6.6	5.9

1 *The Barthel-Index ranges from 0 (fully dependent) to 100 points (fully independent)*.

2 *The PSQI total score (as the sum of all 7 components) ranges from 0 (best sleep quality) to 21 points (worst sleep quality) with a cut-off for bad sleep at ≥6 points*.

3 *The PSQI components 1–7 cover a range of 0 (best result/ no occurence of disturbances) to 3 points (worst result/ most frequent appearance of disturbances) each*.

4 *METH, MET-Hours (Metabolic Equivalent)*. *M, Median; SD, Standard deviation; PSQI, Pittsburgh Sleep Quality Index; METH, MET-Hours*.

### Study Flow

After recruitment, as described above, all participants wore an accelerometer for seven consecutive days. After the 7 days of activity measurement, all participants completed the Pittsburgh Sleep Quality Index (PSQI), MoCA and Barthel Index in an interview. Since physical activity was assessed for the past 7 days and sleep quality for the past 4 weeks at this point, there was a data overlap of 1 week.

### Measurements and Data Processing

The Barthel Index was used for physical disability rating. The questionnaire captures self-reliance in 10 different activities of daily living. The rating works in intervals of five points, starting at 0 going up to 5–15 points depending on the item. Adding up the score of each item, the Barthel-Index ranges from 0 to 100 points. It classifies participants from completely dependent (0) to completely independent (100). Dementia was checked by means of the MoCA. The MoCA is a screening tool for mild cognitive impairment, examining executive function, language abilities, short-term memory and visuospatial processing (28). The cut-off for dementia was a score of <17 points (27).

The amount of physical activity was assessed by means of an accelerometer (MyWellness Key, Technogym, Gambettola, IT). The uniaxial accelerometer captures different intensities of acceleration expressed in a proprietary metric called “Moves.” The different intensities are categorized as “Run” [≥6 MET (Metabolic equivalent)], “Play” (3.0–5.9 MET) and “Free” (1.8–2.9 MET) (29). By means of defined category MET-values, the captured activity was translated into MET-hours prior to further processing. The MyWellness Key exhibits sufficient measurement properties (validity: Pearson's *r* = 0.895–0.944, *p* < 0.001; reliability: ICC > 0.93) (29, 30). All participants were instructed to wear the devices in a horizontal position near the hip for 7 consecutive days. Only accelerometer-datasets of at least 4 completely captured days [10 h wear time each day (31)] were included in the analysis. Datasets of 3 or fewer days were excluded in favor of reliability of the computed average daily activity (31). Accelerometer wear time was captured by a self-administered diary by the participants. Firstly, the proprietary metric of the devices was translated into MET-hours for further analysis. The conversion to MET-values was executed by multiplying the median MET-value of each intensity range with the registered minutes of activity at the corresponding intensity levels (29). Following the WHO definition of sufficient physical activity, a cut-off of 7.5 MET-h/week (150 min = 2.5 h; 2.5 h ^*^ 3MET = 7.5 MET-h) marks the minimum recommended activity level (4). Measurement properties of the accelerometers utilized have been validated satisfactory against indirect calorimetry, as well as crossvalidated for usage in cohorts of older adults (29, 30, 32–34).

Self-reported sleep quality, efficiency, duration and bed rest time were obtained using the Pittsburgh Sleep Quality Index (PSQI). The PSQI captures data on sleep for the last 4 weeks until completion in 19 items (35). The items partially offer open questions (Items 1–4) and partially offer scales, from 0 to 3, mostly defining the frequency of occurrence of sleep complaints (Items 5–14 and 16–17). The total score consists of seven components: (1) subjective sleep quality, (2) sleep latency, (3) sleep duration, (4) sleep efficiency, (5) sleep disturbances, (6) sleeping-pill intake and (7) daytime sleepiness (35). The rating of the subscores is based on the instructions from Smith and Wegener (36), yielding values of 0–3 points for each subscore. The sum of all subscores equals the total score of the PSQI, which is referenced as overall sleep quality hereinafter. The total score ranges from 0 points (highest sleep quality) to 21 points (lowest sleep quality) with a cut-off for poor sleep quality above 5 points (35). Sleep efficiency (Time spent in bed/sleep duration = sleep efficiency) is expressed as a percentage with 100% representing a perfect sleep efficiency. The questionnaire exhibits sufficient reliability (*r* = 0.45–0.84) as well as internal consistency (Cronbach's α = 0.69) (35–37). All PSQI subscores, as well as the total score, were digitalized and checked for plausibility. “Healthy” sleep duration was defined as a period of 6–7 h as this value is found to be an intersection present in the literature (11, 14, 24, 38–40).

### Data Analysis

For statistical analyses, SPSS for Windows (Version 22, IBM, SPSS Inc., Chicago, IL, USA) and BIAS for Windows (Version 9.05, Goethe-University Frankfurt, Germany) were used. A *p*-value of 5% was considered as a relevant cut-off for significance testing. Normal distribution was checked by means of the Shapiro-Wilk-Test (*p* > 0.05). The Levene-Test served for checking homoscedasticity which was given (*p* > 0.05). Linearity was checked by visual inspection. Group differences (sex) were examined by means of *t*-tests for independent samples. Bivariate correlations (Spearman's Rho) were used to explore potential relationships between the amount of physical activity and the sleep quality as PSQI total scores and subscores. In order to minimize confounding factors on physical activity, a subcohort of *n* = 41 participants was created by solely including participants exhibiting a Barthel Index of 90 points or more, free of pre-existing conditions (41). Subsequently, the subcohort was split into four subgroups by the participants' habitual sleep duration. The subgroups were categorized in (1) <5 h of sleep, (2) ≥5 to <6 h of sleep, (3) ≥6 to <7 h of sleep, and (4) ≥7 h of sleep. To analyze differences between the subgroups' physical activity levels univariate ANOVAs were applied, followed by Tukey-HSD *post-hoc* analyses in cases of significance.

## Results

Complete datasets of 64 participants were captured. No participant withdrew his/her consent and no participant was excluded. Participants characteristics are shown in Table 1.

Physical activity levels of the older adults ranged from 3.7 to 35.6 MET-h per week. Only a small proportion met the recommended physical activity levels (3.1%). Participants moved mainly at low-intensity levels (84.5% of overall captured activity), while they were active at medium intensity-levels to a much lesser extent (15.3%) with high-intensity movements being virtually non-existent (0.2%).

The PSQI data show an average total score of 7.2 ± 4.6 points for all participants. Measured scores ranged from 1 to 19 points. Less than half of the cohort (42.2%) can be classified as “good sleepers” by the PSQI total score (≤5 points). Men tended to sleep better according to the present data. Women report a higher incidence in sleep disturbances (Scale of 0 to 3: ♀ = 1.81 ± 1.31 points; ♂ = 1.32 ± 1.43 points; *F* = 1.611, *p* > 0.05), whereas men exhibited higher values regarding sleep efficiency [♀ 76.0 ± 17.7% (range 39.1–100%); ♂ 84.3 ± 11.3% (range 61.1–100%); *p* = 0.026].

**Figure 1:**
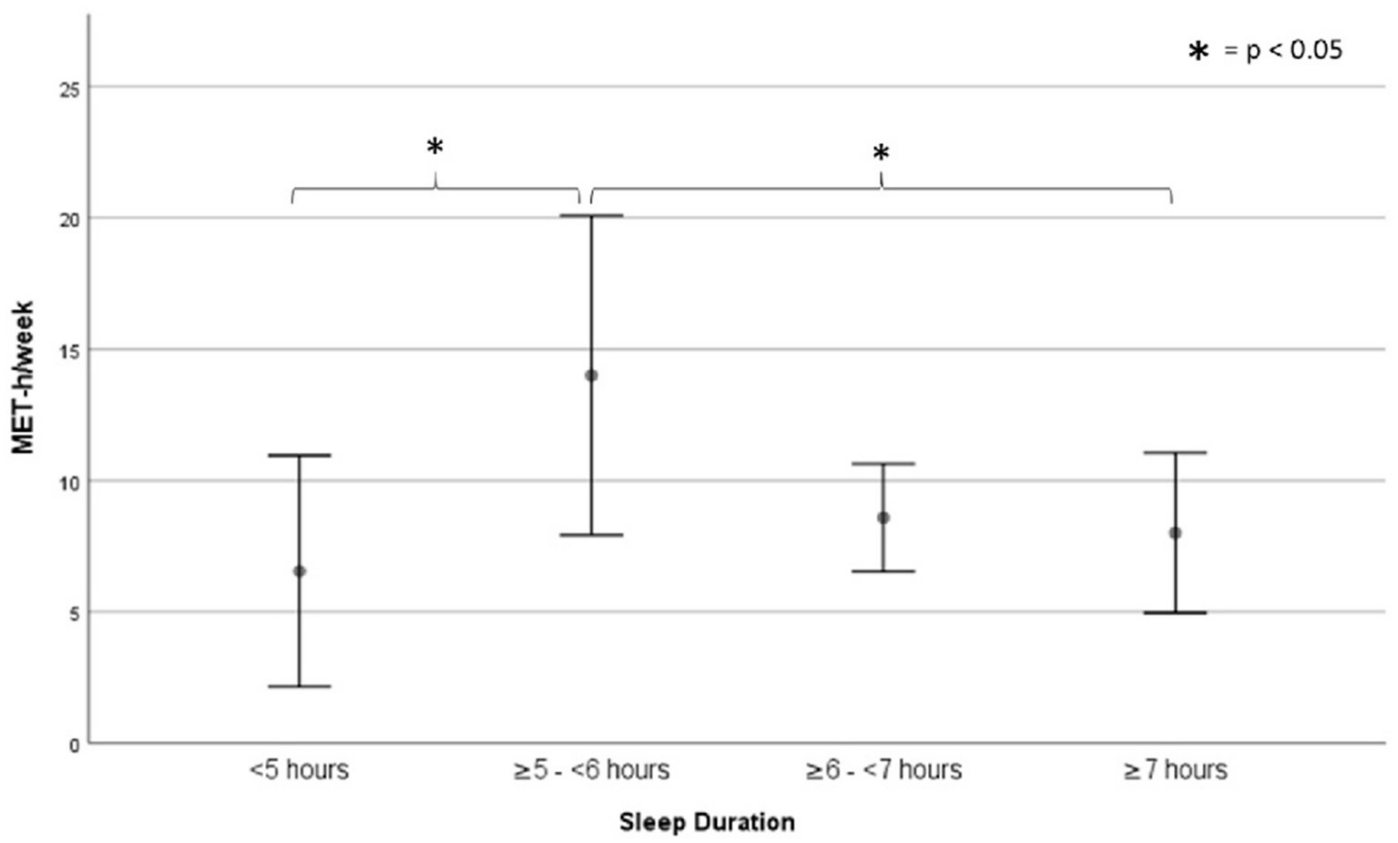
Sub-cohorts' activity levels based on sleep duration subgroups. Data are displayed as means and 95% confidence intervals.

No linear association between physical activity (Accelerometer data) and sleep quality (PSQI total score) was found in the total sample (*r* = 0.119; *p* > 0.05). In the subcohort analysis (only participants with a Barthel Index ≥90 points and free of pre-existing conditions; *n* = 41), the physical activity levels differed significantly (*p* = 0.037) between groups of different sleep duration (Group 1, *n* = 7, <5 h of sleep; Group 2, *n* = 11, ≥5 to <6 h of sleep; Group 3, *n* = 10, ≥6 to <7 h of sleep; Group 4, *n* = 13, ≥7 h of sleep) (Figure 1). More precise, the average MET-hours per week were 13.5 ± 7.4 (M ± SD) for participants sleeping <5 h (Group 1), 23.5 ± 9.7 for participants sleeping up to 6 h (Group 2), 16.8 ± 4.3 for participants sleeping up to 7 h (Group 3) and 15.0 ± 7.5 for participants sleeping 7 h or more (Group 4).

Participants sleeping ≥5 to <6 h/night differed from participants exhibiting other sleep durations (≥7, ≥6 to <7, and <5 h) in the activity per week (*p* = 0.005) and in the sleep efficiency (*p* < 0.05).

## Discussion

Habitual physical activity and sleep duration appear to be linked in the present cohort. However, we found no accordance between higher activity levels and better sleep quality in the investigated cohort *per se*. A self-reported sleep duration of ≥5 to <6 h, corresponding to 7.6 h bed rest time, is, however, associated with a higher level of physical activity.

Besides low activity levels among the cohort, the measured intensity values align with the values determined by a similar study (42). Only 3.1% of the cohort reached the recommended extent of physical activity above an intensity of ≥3 MET. Since the MyWellness Key calculates “Moves” solely from captured acceleration, regardless of sex, age, weight and height of the wearer, we hypothesize that there is some discrepancy between measured and experienced exercise intensity. The accelerometer data seem to underestimate the intensity experienced by our geriatric cohort. The need to adjust the accelerometer data for the intensity experienced in older adults has already been addressed in the literature (43, 44). Even though there is disagreement among the literature whether accelerometry reliably reflects experienced intensity in older adults, there are indications that the generalized limits of the intensity levels need age-appropriate cut-off points. For instance, even a slow walking speed (1.5 mph) was associated with moderate-intensity energy expenditure in a cohort of older adults (45). Contrary to the accelerometer's raw data, we thus assume our participants to be more active than equivalent-aged reference populations.

Our findings regarding a higher incidence of sleep disturbances and lower sleep quality in women align with related studies' results (46–48).

There is agreement on the health risks caused by “too” long or short sleep durations, such as increased all-cause mortality, cardiovascular mortality and lower cognition capability (39, 40). The discussion regarding healthy sleep duration remains controversial; estimates vary from 3 to 5 h as being too short, while 8–10 h is regarded as being too extended for sleep duration (38, 49). By reporting an average sleep duration of 6.7 ± 1.8 h, the present cohort shows a healthy sleep duration, irrespective of the quoted source.

The fact that the present cohort almost exclusively moved at lower intensity levels assumingly contributes to the missing correlations between physical activity and sleep parameters. It is feasible that there may still be a relationship between higher intensity activity and sleep parameters that has remained concealed in the present study. Despite the existing relationship of low intensity activity to health, an explanation for any missing correlation of low intensity activity with sleep parameters is absent in the literature (18, 50, 51).

However, regardless of the intensity levels, the present data indicate a trend towards a relationship between activity extent and self-reported sleep duration. Participants reporting ≥5 to <6 h of sleep move more than any group reporting different sleep durations and, consequently, when taking activity of all intensity levels into account, are more likely to be sufficiently physically active in terms of current guidelines. As majoritarian literature quantifies “healthy” sleep duration as ≥6 to ≤7 h (38, 49, 52), the most advantageous sleep duration may diverge, depending on the underlying criteria. Consequently, the sleep duration resulting in the greatest health benefits may not be the same as the one resulting in the highest amount of physical activity. Furthermore, sleep duration is assumed to depend on physical activity behavior (53), leading to a presumably mutual interference of both. The assumption that the combination of shorter sleep durations with higher activity levels is derived from gained time to move is refuted by the similar overall bedtimes adopted across all activity groups. Additionally, the most active group exhibits the lowest sleep efficiency; this highlights that increased physical activity is not, *per se*, a warrantor for high sleep quality. All in all, present data indicate an inverted U-shaped curvilinear relationship of physical activity and sleep duration.

Present study is not free of limitations. Regarding the findings of our statistical analysis a larger sample size would consolidate validity and generalizability of our results. Furthermore, an additional objective measurement of sleep parameters would enhance data quality compared to a solely subjective assessment tool. Measurements of physical activity may gain additional quality from usage of multiaxial accelerometers over the uniaxial instruments used in present investigation. Some modern devices allow the measurement of both, physical activity and sleep, within a single instrument.

The dominant direction of influence is still questionable in the mutual interference between sleep and physical activity. The questions: “Which of these two factors has the stronger impact on health?” and “Are these factors combinable for a maximum benefit on old age health status?” still remain. To explore these health-relevant issues, future studies should address both factors and, ideally, should also assess the direction of the causal connection. We recommend a cohort exhibiting corresponding activity levels in order to analyze the relationship of higher intensity activity with sleep parameters. For this purpose, it might be appropriate to recruit participants e.g., in sports clubs and sports facilities rather than from general population. Besides existing recommendations on physical activity, the implementation of recommendations and information on healthy sleep behavior might benefit older adults. Since both affect health over the long term, raising awareness to optimize physical activity and sleep in this particular population group, may be of more value than brief interventions.

## Conclusion

Even if there is no linear association between sleep quality and physical activity, both factors appear to be linked. Differing activity levels between groups of various self-reported sleep durations indicate a coherence of sleep and activity extents. A deeper understanding of these factors' relationship may enable research on the compatibility of their health benefits and, therefore, contribute to the improvement of general and geriatric health promotion.

## Data Availability Statement

The datasets generated and analyzed during the current study are available from the corresponding author on reasonable request.

## Ethics Statement

The studies involving human participants were reviewed and approved by Ethics Committee, Goethe-University Department 05, Faculty of Psychology and Sport Sciences. The patients/participants provided their written informed consent to participate in this study.

## Author Contributions

LV, OV, and IE-R: study concept and design. OV and IE-R: acquisition of data. DN and JW: analysis and interpretation of data. OV, DN, JW, IE-R, and LV: preparation of manuscript. All authors have read and approved the manuscript.

## Conflict of Interest

The authors declare that the research was conducted in the absence of any commercial or financial relationships that could be construed as a potential conflict of interest.

## Publisher's Note

All claims expressed in this article are solely those of the authors and do not necessarily represent those of their affiliated organizations, or those of the publisher, the editors and the reviewers. Any product that may be evaluated in this article, or claim that may be made by its manufacturer, is not guaranteed or endorsed by the publisher.

## Abbreviations

ADL: Activity of Daily Living
ICC: Intra Class Correlation
M: Mean
MET: Metabolic Equivalent
MoCA: Montreal Cognitive Assessment
PSQI: Pittsburgh Sleep Quality Index
REM-sleep: Rapid Eye Movement-sleep
SD: Standard Deviation
WHO: World Health Organization.
